# Use of acupuncture to reduce prolactin levels in antipsychotically induced hyperprolactinemia: a case report

**DOI:** 10.3389/fmed.2026.1727171

**Published:** 2026-05-14

**Authors:** Qi Wang, Zhihui Hou, Yuqi Jia, Meina Wang, Zhaozhan Xie, Hongling Jia

**Affiliations:** 1 The Second Affiliated Hospital of Shandong University of Traditional Chinese Medicine, Jinan, Shandong, China; 2 School of Acupuncture-Moxibustion and Tuina, Shandong University of Traditional Chinese Medicine, Jinan, Shandong, China

**Keywords:** acupuncture, case report, drugs, hyperprolactinemia, schizophrenia

## Abstract

**Introduction:**

Hyperprolactinemia, an endocrine disorder, has witnessed a notable increase in incidence in recent years, exerting a substantial influence on patients’ physiological states and quality of life. Hyperprolactinemia has been detected in 65% of women of childbearing age and 40.70% of men using antipsychotic medications. The extent to which antipsychotic drugs cause elevated serum prolactin levels primarily depends on their affinity for D2 receptors and their ability to cross the blood–brain barrier. The stronger their binding to D2 receptors and the poorer their ability to cross the blood–brain barrier, the greater the elevation in serum prolactin levels. Consequently, the management of hyperprolactinemia plays a pivotal role in enhancing treatment adherence, quality of life, and long-term health outcomes among patients with schizophrenia. Although some studies have confirmed the efficacy of acupuncture in treating hyperprolactinemia induced by antipsychotic drugs, more specific studies are needed.

**Case:**

A 30-year-old woman with hyperprolactinemia presents with scanty and prolonged menstrual cycles, urinary leakage, behavioral abnormalities, and difficulty concentrating. She had a history of undifferentiated schizophrenia. Acupuncture was used for 3 weeks, with three treatments per week, each time leaving the needles in place for 30 min.

**Results:**

After 3 weeks of acupuncture treatment, this patient had increased menstrual flow, normalization of leucorrhea, a marked improvement in mental status, a decrease in abnormal behavior, and a decrease in body weight from 82 kg to 72 kg, and no further urinary leakage. After 44 days, the prolactin level decreased from the initial 192.42 ng/mL to 146.53 ng/mL, the Positive and Negative Syndrome Scale (PANSS) score decreased from an initial score of 91 to 52, the menstrual cycle and vaginal discharge are normal, and body weight decreased to 62 kg.

**Conclusion:**

This case study suggests that acupuncture may improve the mental well-being of individuals with psychiatric conditions and concurrent hyperprolactinemia, reduce prolactin levels, improve menstrual flow and cycle, and decrease urinary incontinence. Therefore, acupuncture may have potential benefits in the treatment of antipsychotic-induced hyperprolactinemia.

## 1 Case presentation

A 30-year-old woman has exhibited a withdrawn personality for more than 10 years, with her symptoms worsening and accompanied by irregular menstruation for the past 5 months. With family history of schizophrenia, she was diagnosed with “undifferentiated schizophrenia” and hospitalized on 22 December 2020 at the Shandong Mental Health Center, where she was given oral quetiapine fumarate tablets (0.1 g, qn), trihexyphenidyl tablets (2 mg, bid), amisulpride tablets (0.4 g, bid), psychotherapy, and transcranial therapy. After discharge, the patient were prescribed the following medications: quetiapine fumarate tablets (0.1 g, qn), trihexyphenidyl tablets (2 mg, bid), and amisulpride tablets (0.4 g, bid). One month after taking the medication, she had low menstrual flow, long menstrual cycle, abundant and thick leukorrhea, leakage of urine, and abnormal behavior, which was manifested by occasional rolling of white eyes, licking of incisors, sucking of teeth, spitting, and rejecting the care of family members. In order to seek systematic treatment, the patient visited the Acupuncture Department of the Second Affiliated Hospital of Shandong University of Traditional Chinese Medicine on 1 July 2021. Physical examination revealed that the patient weighed 82 kg. PANSS score of 91. A urine pregnancy test was negative. Thyroid function tests (TSH, FT4) were within normal limits. Renal and liver function panels were unremarkable. On 1 July 2021, prolactin content and cranial CT examination were performed, and the prolactin content was 192.42 ng/mL (Table 1), with no obvious abnormal density shadows in the brain parenchyma, no obvious abnormality in the ventricular system, and the sulcal gyrus was clearly displayed, with a centered midline structure. The patient denied taking any other medications besides the aforementioned drugs. She reported having stable sleep patterns and no work-related stress, which remained unchanged during the treatment. There was no history of recent vigorous exercise or breast stimulation prior to blood draws.

**Table 1:** Change of prolactin level (Laboratory Report of Shandong Mental Health Center).

Serum prolactin determination (chemiluminescence method)					
No.	Code	Item	Result	Unit	Reference range
Sampling time: 2021-7-1 8:50					
1	PRL	Prolactin	192.42↑	ng/mL	3.8–30.7
Sampling time: 2021-9-4 9:50					
1	PRL	Prolactin	146.53↑	ng/mL	3.8–30.7

## 2 Past medical history

The patient was diagnosed with depression in middle school due to emotional problems, and her depression worsened in 2019 due to family changes, and she was diagnosed with “undifferentiated schizophrenia” on 22 December 2020 at the Shandong Mental Health Center.

## 3 Interventions

The authors utilized acupuncture to treat the patient three times per week. The acupuncture points are illustrated in Table 2. *Operational methods* (1, 2): The patient was positioned in the prone position, and the posterior neck was routinely sterilized using 75% alcohol. Disposable sterile acupuncture needles of 0.25 mm × 40 mm were selected. The needles were inserted sequentially from the right to the left, insert the Xiangqi needles points sequentially: GB12 (bilaterally), GB20 (bilaterally), BL10 (bilaterally), GV16. Twisting was performed during needling to facilitate the flow of qi, the amplitude was maintained within 180 degrees, and the frequency was set at 60 times per minute. LR3 and L14 acupoints were treated with twisting drainage method, while SP6 acupoint was treated with twisting tonification method. Once the bring about the desired sensation had been obtained, the twisting was stopped and the needle was left in place for a period of 30 min. The treatment was administered three times per week for a total of 6 weeks, with no changes in the types or dosages of oral medications during the treatment period. No adverse reactions occurred from the acupuncture.

**Table 2:** Points used with direction, depth of placement, and intended actions.

Point	Direction	Depth (mm)	Intended action
Feng Fu (GV16)	Skew toward the chin	15 ~ 25	Unifies the yang, regulates the medulla oblongata, and generates yang energy in the five organs.
Feng Chi (GB20)	Skew toward the tip of the nose	20 ~ 25	Harmonize the Yin and Yang of the five viscera
Tian Zhu (BL10)	Perpendicular	15 ~ 25	Benefiting the essence and filling the marrow, calming the mind and promoting the flow of qi and blood in the head
Wan Gu (GB12)	Perpendicular	20	Relaxing the liver and regulating qi, relieving depression and regulating the mind, clearing the head and eyes
Tai Chong (LR3)	Perpendicular	13	Clearing away heat and dispersing evil spirits, detoxifying the liver and catharticizing fire
He Gu (L14)	Perpendicular	13	Clearing away heat and dispersing evil spirits, detoxifying the liver and catharticizing fire
San Yin Jiao (SP6)	Perpendicular	13	invigorate the liver, strengthen the spleen and tonify the kidneys

The Xiangqi needles Acupuncture Therapy is one of the distinctive techniques of Qilu Traditional Chinese Medicine, which has been inherited and developed over six generations. It is widely applied in the treatment of diseases related to internal medicine, surgery, gynecology, pediatrics, and psychiatry, demonstrating significant clinical efficacy (3). The Xiangqi needles Acupuncture Therapy involves seven needles at four acupoints: Fengfu (BL17), Tianzhu (BL20), Fengchi (BL20), and Wanggu (BL20). This therapy is characterized by dredging the channel, regulation of qi and blood, calming the mind and strengthening the will, filling of the pulp, regulate qi movement (3).

## 4 Discussion

Hyperprolactinemia, an endocrine disorder, has witnessed a notable increase in incidence in recent years, exerting a substantial influence on patients’ physiological states and quality of life. Hyperprolactinemia has been detected in 65% of women of childbearing age and 40.70% of men using antipsychotic medications (4). Hyperprolactinemia is a relatively common adverse reaction during the use of antipsychotic drugs in clinical practice. It can lead to disorders of the hypothalamic–pituitary-gonadal axis, and its clinical manifestations often include infrequent menstruation, amenorrhea, galactorrhea, and infertility (5, 6). The extent to which antipsychotic drugs cause elevated serum prolactin levels depends primarily on their affinity for D2 receptors and their ability to cross the blood–brain barrier; the easier they bind to D2 receptors, the harder they cross the blood–brain barrier, and the greater the elevation in serum prolactin levels (7). Consequently, the management of hyperprolactinemia plays a pivotal role in enhancing treatment adherence, quality of life, and long - term health outcomes among patients with schizophrenia. Although some studies have confirmed the efficacy of acupuncture in treating hyperprolactinemia induced by antipsychotic drugs, more specific studies are needed (8, 9). This case report describes the successful treatment of a patient with undifferentiated schizophrenia with hyperprolactinemia and illustrates many key points applicable to clinical practice. In the initial treatment, acupuncture may help to restore normal menstrual flow and leukorrhea, normalize leukorrhea, reduce urine leakage, regulate prolactin levels, and regulate gynecological disorders. Acupuncture may also be associated with improvements in the patient’s mental state and abnormal behaviors, demonstrating that acupuncture can treat psychiatric disorders to some extent. Patient lost weight during treatment, indicating that acupuncture may promote the body’s metabolism. The combination of Xiangqi needles and other acupuncture points may be the key.

While the observed reduction in serum prolactin (from 192.42 ng/mL to 146.53 ng/mL) is substantial, it is important to note that the level remained significantly above the normal reference range for females. This finding implies that acupuncture could function as an adjunctive therapy instead of a substitute for conventional endocrine treatment. This also underscores the necessity of implementing a multidisciplinary diagnosis and treatment approach, which encompasses endocrinology consultation. The clinical meaningfulness of such a reduction must be interpreted with caution. However, even a partial reduction can be beneficial, as it may alleviate the severity of antipsychotically induced hyperprolactinemia related symptoms (e.g., amenorrhea, galactorrhea) and potentially reduce long-term risks. Furthermore, while the antipsychotic regimen was stable, we cannot rule out the influence of other unmeasured factors that may have coincided with the acupuncture treatment. These could include natural hormonal fluctuations, changes in stress levels, sleep patterns, or diet, which might have contributed to the observed changes in prolactin. Although the initial CT scan results in this case showed no abnormalities, the significantly elevated serum prolactin level (192 ng/mL) was classified as atypical hyperprolactinemia. MRI serves as a superior imaging modality for excluding pituitary adenomas, which constitutes a critical learning point in this case. It is recommended that MRI should be employed as a necessary diagnostic tool in similar scenarios.

## 5 Conclusion

The findings in this case suggest that the use of acupuncture may reduce prolactin levels in hyperprolactinemia, and although this hyperprolactinemia was caused by antipsychotics, it may be equally informative for other causes of hyperprolactinemia. This case demonstrates positive therapeutic effects, but further studies are required to confirm the therapeutic efficacy of acupuncture, such as randomized, double-blind, sham acupuncture-controlled trials.

## Data Availability

The original contributions presented in the study are included in the article/supplementary material, further inquiries can be directed to the corresponding author.
